# Bardet-Biedl syndrome with end-stage kidney disease in a four-year-old Romanian boy: a case report

**DOI:** 10.1186/1752-1947-5-378

**Published:** 2011-08-15

**Authors:** Cristina M Mihai, Jan D Marshall, Ramona M Stoicescu

**Affiliations:** 1 Faculty of Medicine, "Ovidius" University, 145 Tomis Blvd, Constanta 900591, Romania; 2 The Jackson Laboratory 600 Main Street Bar Harbor, Maine 04609 USA; 3 Faculty of Pharmacy, "Ovidius" University, 145 Tomis Blvd, Constanta 900591, Romania

## Abstract

**Background:**

Bardet-Biedl syndrome is a significant genetic cause of chronic kidney disease in children. Kidney abnormalities are a major cause of morbidity and mortality in Bardet-Biedl syndrome, but the onset of end-stage renal disease at an early age and continuous ambulatory peritoneal dialysis, however, are not commonly mentioned in the literature.

**Case presentation:**

We present the case of a four-year-old Romanian boy who presented to our department with 'febrile seizures'. After an initial evaluation, we diagnosed our patient as having hypertension, severe anemia and end-stage renal disease. He met the major and minor criteria for the diagnosis of Bardet-Biedl syndrome and underwent continuous ambulatory peritoneal dialysis.

**Conclusions:**

Close follow-up for renal involvement in patients with Bardet-Biedl syndrome and Alström syndrome from an early age is highly recommended to prevent end-stage renal disease and so renal replacement therapy can be started immediately.

## Introduction

Chronic kidney disease is an irreversible condition that eventually progresses to end-stage renal disease (ESRD). In children, this can be the result of heterogeneous diseases of the kidney and urinary tract ranging from common congenital malformations of the urinary tract, to rare diseases that affect kidney function. ESRD is an important cause of morbidity and mortality in children worldwide [1,2].

Bardet-Biedl syndrome (BBS) is a rare, genetic multisystem disorder; a ciliopathy secondary to the basal body dysfunction [3,4]. Mutations in 14 genes are known to be associated with BBS: *BBS1*, *BBS2*, *ARL6/BBS3*, *BBS4*, *BBS5*, *MKKS/BBS6*, *BBS7*, *TTC8/BBS8*, *B1/BBS9*, *BBS10*, *TRIM32/BBS11, BBS12, MKS1/BBS13*, and *CEP290/BBS14 *[5]. BBS shares many similarities with Alström syndrome, caused by mutations in the gene *ALMS1 *and inherited in an autosomal recessive manner, but Alström syndrome (ALMS) is characterized by relative preservation of cognitive function and the absence of polydactyly [6].

Due to the genetic heterogeneity diagnosis of BBS primarily relies on clinical findings and family history. This pleiotropic disorder has variable expressivity and a wide range of clinical variability observed both within and between families [7]. The main clinical features are rod-cone dystrophy with childhood-onset night blindness and visual loss, post-axial polydactyly, truncal obesity that manifests during infancy and remains problematic throughout adulthood, specific learning difficulties, male hypogenitalism and complex female genitourinary malformations. Chronic renal dysfunction resulting from kidney abnormalities is a major cause of morbidity and mortality [8].

Conventional approaches to end-stage renal disease in such patients are chronic peritoneal dialysis and hemodialysis followed by kidney transplantation. Continuous ambulatory peritoneal dialysis, however, is not a commonly advocated modality in the literature.

## Case presentation

A four-year-old Romanian boy was admitted to Spitalul Clinic Judetean de Urgenta Constanta, Romania for 'febrile seizures'. An initial evaluation revealed hypertension, severe anemia and end-stage renal disease.

**Figure 1:**
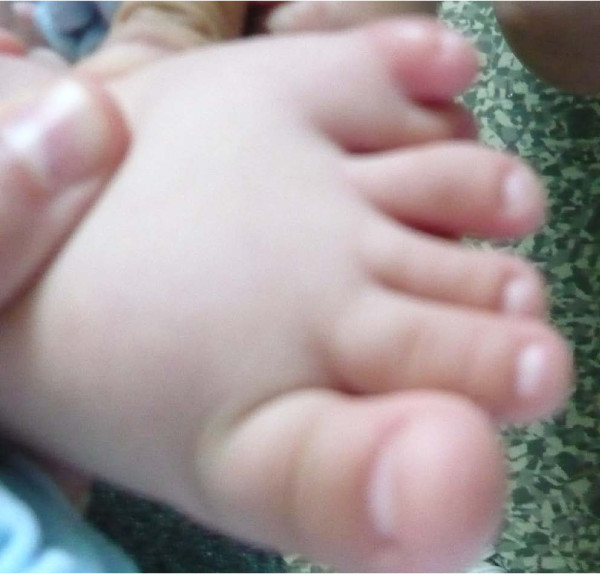
**Polydactyly of the fifth finger before surgery**.

Our patient is the second offspring of consanguineous parents. His family history was notable for obesity, learning difficulties, six digits on two limbs and visual impairment in his 14-year-old sister. He had also six digits on two limbs, diagnosed at birth (Figure 1). No ultrasonography was performed during the pregnancy or during the neonatal period. Initial motor and mental development milestones were abnormal, a delay noted by the family doctor, but the diagnosis was not established in the context of family history and consanguinity of the parents.

**Figure 2:**
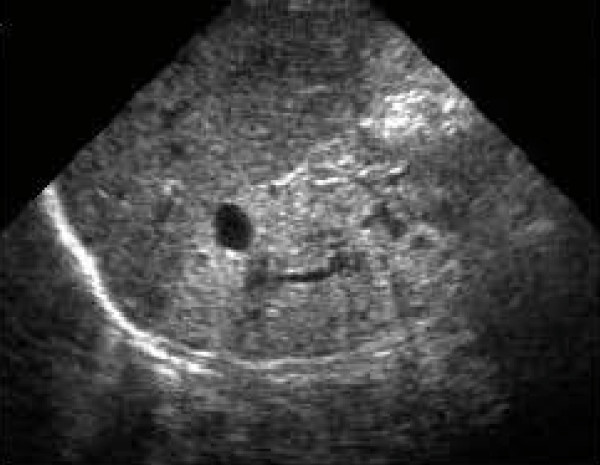
**Renal ultrasonography**. Bilateral renal enlargement and increased renal parenchymal echogenicity.

Nystagmus and pigmentary retinopathy, mild central obesity, male hypogonadism (microtestis and microphallus on genital examination), mental retardation, behavioral abnormalities, hypothyroidism, and abnormal dentition were observed. Digital abnormalities included partial syndactyly (between the second and the third fingers), fifth finger clinodactyly of both hands, brachydactyly of both hands and feet, and minor scars after surgical removal of the sixth digit of the left hand and left foot. Renal involvement was very mild. Bilateral renal enlargement and increased renal parenchymal echogenicity (Figure 2) were the early findings of imaging studies performed at two years old, whereas urea and creatinine values were slightly elevated (45 mg/dL and 1.4 mg/dL). Hypertension, type 2 diabetes mellitus, congenital heart disease, hearing impairment or cardiomyopathy were not identified at the first consultation.

According to the clinical and paraclinical evidence, he met all of the six cardinal or primary criteria and six minor or secondary criteria (Table 1) necessary for a diagnosis of BBS [7]. His older sister was also diagnosed with BBS at the same time, based on clinical assessment. No genetic testing for BBS was available at our hospital.

**Table 1:** Diagnostic criteria in Bardet-Biedl syndrome (BBS)

Primary features of BBS	Secondary features of BBS
**Retinal dystrophy**	**Developmental delay**
**Post-axial polydactyly**	**Behavioral problems**
**Obesity**	Neurological problems
**Hypogenitalism**	**Speech disorder**
**Renal abnormalities**	**Brachydactyly, syndactyly, or clinodactyly**
**Learning disabilities**	**Dental anomalies**
	Nephrogenic diabetes insipidus
	Diabetes mellitus
	**Hypertension**
	Anosmia

Features in our patient are shown in bold.

The mother and the family doctor were advised to check his renal status routinely; the boy was not seen for routine medical controls for two years, until he suddenly developed an episode of a generalized tonic-clonic seizure. He was admitted to the local health facility and was found to have hypertension, severe anemia (hemoglobin 6 g/dL) and renal impairment (serum creatinine 8.5 mg/dL).

**Figure 3:**
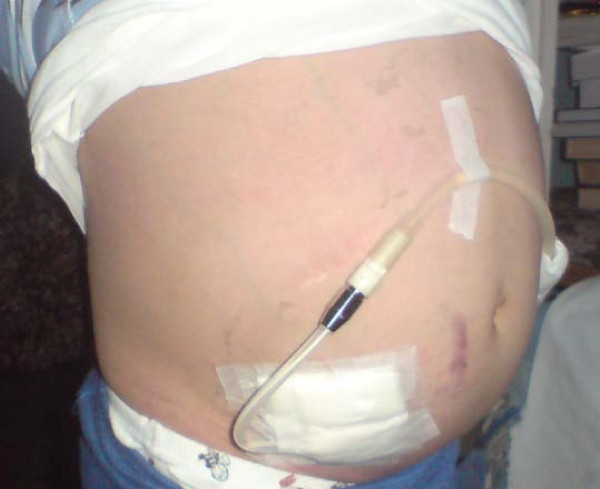
**Our index patient with peritoneal dialysis catheter**.

A diagnosis of BBS with ESRD was made. Due to the elevated urea and serum creatinine levels accompanied by uremic symptoms (loss of consciousness and generalized seizures), a Tenckhoff catheter was inserted and he was initiated on daytime ambulatory peritoneal dialysis (Figure 3).

After a 15-month follow-up, his evolution was satisfactory without any complications with the catheter. His glomerular filtration rate was stabilized.

Our patient's 14-year-old sister was not obese, and had polydactyly in one hand and one foot. Rod-cone dystrophy with myopia and strabismus was diagnosed at eight years of age. Neurodevelopmental delay had been noted since infancy, and evolved with hypotonia, dyspraxia, poor balance, and ataxic gait. Speech and behavior problems were noted, as well as learning difficulties. No renal tract abnormalities were diagnosed. Dental anomalies, such as crowding of the teeth, hypodontia, and high arched palate were noticed. She has a typical facies with enophthalmos and downward slanting palpebral fissures.

Another boy, a third degree cousin in the same family, was diagnosed with mild obesity, insulin resistance, glucose intolerance, then diabetes mellitus, hypertension, dyslipidemia, renal dysfunction, and cardiac involvement (dilated cardiomyopathy). This patient died due to a myocardial infarction at 28 years of age.

He also had a brother who died at nine months of age, presenting with signs and symptoms suggestive for cardiac failure. Both of these patients presented with nystagmus starting from four months of age (Table 2).

**Table 2:** Comparison between our index patient and his cousin

Index patient	His cousin
Nystagmus in early infancy	Nystagmus and photodysphoria in early infancy
Rod-cone dystrophy, diagnosed at three years of age	Progressive pigmentary retinopathy (rod-cone dystrophy) leading to blindness
Post-axial polydactyly, hexadactyly of the fifth finger in one hand and foot	No light perception by age 14 years
Mild central obesity	Normal extremities/absence of polydactyly or syndactyly
Hypogonadism and hypogenitalism	No childhood obesity, normal weight in adulthood
Renal dysfunction (end-stage renal disease) diagnosed at age 4	Normal genitalia
Mental retardation	Progressive chronic nephropathy, chronic renal failure
One sister with a similar phenotype, but without renal impairment at 14 years of age	Normal intelligence
	Mild to moderate bilateral sensorineural hearing loss
	Congestive heart failure secondary to dilated cardiomyopathy in early adulthood (severe)
	Hyperinsulinemia/insulin resistance
	Non-insulin dependent diabetes mellitus developed in early adolescence
	Elevation of hepatic enzymes
	Hepatic steatosis
	Hepatic dysfunction
	Alopecia
	Low levels of growth hormone
	Short stature
	Advanced bone age
	Hypertension
	Hyperlipidemia
	Hypertriglyceridemia
	Atherosclerotic disease (aorta)
	Hyperuricemia
	Hypersecretory lungs
	One brother who died in infancy with signs and symptoms suggestive for cardiac failure

**Figure 4:**
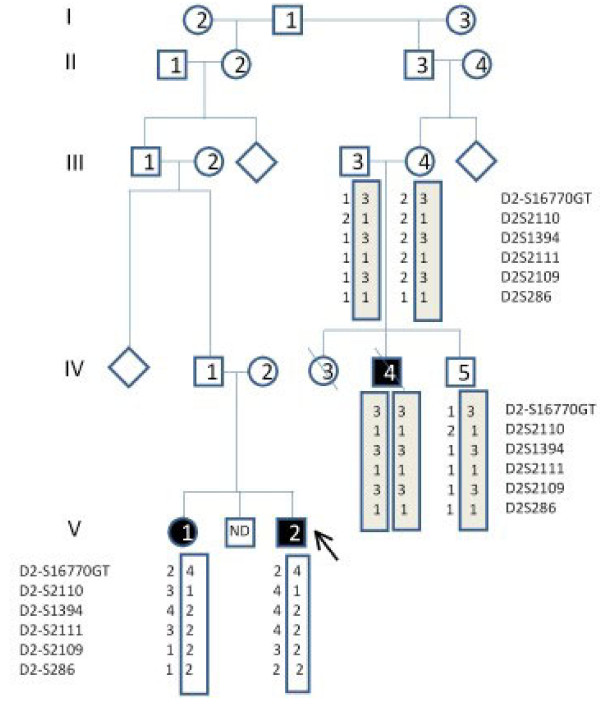
**Pedigree and haplotype**. Our index patient (1) and his sister (2) share only one halplotype in the Alström region. Additionally, the haplotype they share is not the same as their cousins (3-5). Our patient's cousin (4) and patient's sister (2) share a single nucleotide polymorphism (non-disease causing) in Bardet-Biedl syndrome 5 (BBS5), which was probably passed down from their grandfather. It is not really relevant, because it is not causative. Our patient's cousin is homozygous in the *ALMS1 *region and his brother is a 'carrier', which suggests a diagnosis of Alström syndrome could be correct.

Our patient's cousin's symptoms are more compatible with a diagnosis of Alström syndrome, while the siblings are closer to a diagnosis BBS. Our patient, his sister and their cousin were included in a mutation analysis (Jackson Laboratory, Bar Harbor, Maine) and were screened using a microarray (Asper Ophthalmics, Tartu, Estonia) containing 155 published disease-causing variants and polymorphisms in 11 BBS genes and 98 in *ALMS1*. Our patient and his cousin shared a heterozygous single nucleotide polymorphism in BBS5 (BBS5_N184S het) that is not disease causing. Haplotype analysis showed that our patient and his sister shared only one allele in the ALMS1 region, meaning that the disorder shared by them is most likely not Alström syndrome (Figure 4). Efforts are still being made to identify possible novel mutations that may explain the phenotypic diagnosis.

## Discussion

Recent findings in genetic research have suggested that a large number of genetic disorders that were not previously identified in the medical literature as associated, may, in fact, be highly related through the primary cilia. Cilia are small, hair-like appendages attached to the surface of human cells. They act like antennae, sensing and evaluating extracellular signals to coordinate the development and stability of a wide variety of organs. Ciliopathies are a newly emerging group of genetic diseases caused by defects in the function or structure of cellular primary cilia. These diseases present with symptoms such as mental retardation, retinal blindness, obesity, polycystic kidney disease, liver fibrosis, ataxia and some forms of cancer. Thus, BBS is a ciliopathy; a rare, multi-tissue disorder linked to mutations in 14 different proteins. The pleiotropic phenotype is due to dysfunction of basal bodies and cilia [9,10].

The variable manifestations of BBS were initially described by Bardet and Biedl in the 1920s [11]. Renal dysfunction has been recognized only recently to be a component of the BBS clinical phenotype. Renal malformations in BBS had been reported infrequently, although a high frequency of structural abnormalities were observed post-mortem. In one study, 26 of 57 patients (46%) had renal structural abnormalities. However, only 5% had functional impairment at the time of assessment [7,11].

Somwanshi reported on four cases (three males, one female) with polydactyly, hypogonadism, retinitis pigmentosa, obesity, and mental retardation; however, renal function was normal in all of these cases [12]. Pal and Bhattacharyya described an 18-year-old woman with pigmentary retinopathy, hypogenitalism, dwarfism, polydactyly, obesity, and mental retardation, but without renal involvement [13]. Cysts in the left kidney were detected in a 30-year-old patient; her renal function, however, was normal [14]. Gupta [15] reported a 20-year-old woman with renal insufficiency and multiple fractures, possibly related to renal osteodystrophy; her serum creatinine level was 3.0 mg/dL and ultrasonography revealed bilateral hypoplastic kidneys. Rathi described the first case from India with ESRD who was treated with continuous ambulatory peritoneal dialysis [16]. Hooda *et al. *described the case of a 12-year-old boy diagnosed as having BBS with stage III chronic kidney disease that progressed to ESRD with consistent creatinine of approximately 11 mg/dL and calculated glomerular filtration rate of 5.68 mL/minute. He underwent successful renal transplantation [17].

The frequency of renal involvement reported in BBS varies. A questionnaire-based evaluation [7] reported anatomic anomalies, renal insufficiency, and ESRD, with abnormalities including renal cysts, fetal lobulation, scarring, dysplasia, unilateral agenesis, ectopia, vesico-ureteric reflux, and calyceal clubbing or blunting. Renal insufficiency is noted in approximately 5% to 25% of patients with BBS, progressing to ESRD in 4% to 10%. Renal failure is the commonest cause of death in BBS [16-18]. Renal histology has revealed chronic interstitial nephritis, mesangial proliferative glomerulopathy, and ultrastructural changes in the glomerular basement membrane [19,20]. Most cases of BBS are diagnosed after the first decade of life and diagnosis in early childhood is very rare unless there is a family history [7,10].

Our patient's case was particularly interesting in that he presented with ESRD at an earlier age than the most other reported cases. The delay of diagnosis in the context of consanguinity of the parents and the existence of similar cases in the family is surprising, but could be explained by the reluctance of this family to provide sufficient data to the doctors regarding the other affected members. Also, ultrasonography during pregnancy is not compulsory in Romania and this could explain why the patient's kidneys were not assessed before birth. Even after birth, no connection was made between the hexadactily and a possible genetic disorder.

Although the BBS/ALMS APEX array improves the mutation detection possibilities of first-line mutation screening, it is clear that some mutations that contribute to these diseases remain unknown, mainly in less studied populations. It is essential that future research endeavors determine the prevalent mutations in such populations, followed by inclusion of newly identified mutations on the BBS/ALMS1 APEX chip (or other diagnostic tests), so that appropriate genetic diagnosis, counseling, and understanding of the pathogenesis and outcomes of these diseases can be achieved in all ethnic groups.

The management of renal failure in BBS does not differ from that due to any other cause and all three modalities of long-term renal replacement therapy (RRT), that is, hemodialysis, chronic peritoneal dialysis, and renal transplantation can be offered to these individuals. Nonetheless, it represents a very rare indication for kidney transplantation.

## Conclusions

Close follow-up for renal involvement in patients with BBS and ALMS from an early age is highly recommended to prevent ESRD and also so renal replacement therapy can be started immediately.

## Consent

Written informed consent was obtained from the patient's next-of-kin and the patient's cousin's next-of-kin for publication of this case report and any accompanying images. A copy of the written consent is available for review by the Editor-in-Chief of this journal.

## Competing interests

The authors declare that they have no competing interests.

## Authors' contributions

RMS was responsible for analyses of blood samples and initial drafting of the manuscript. JDM was responsible for the genetic testing, planning and drafting the manuscript. CMM was responsible for design, planning, execution and drafting of the manuscript. All authors read and approved the final manuscript.
